# Pharmacokinetic Characteristics of Siponimod in Healthy Volunteers and Patients With Multiple Sclerosis: Analyses of Published Clinical Trials

**DOI:** 10.3389/fphar.2022.824232

**Published:** 2022-05-10

**Authors:** Chen Chaoyang, Dong Xiu, Wei Ran, Ma Lingyun, Zhao Simiao, Li Ruoming, Zhang Enyao, Zhou Ying, Cui Yimin, Liu Zhenming

**Affiliations:** ^1^ Department of Pharmacy, Peking University First Hospital, Beijing, China; ^2^ Department of Pharmacy Administration and Clinical Pharmacy, School of Pharmaceutical Science, Peking University, Beijing, China; ^3^ Institute of Clinical Pharmacology, Peking University, Beijing, China; ^4^ State Key Laboratory of Natural and Biomimetic Drugs, School of Pharmaceutical Sciences, Peking University Health Science Center, Beijing, China

**Keywords:** model-based meta-analysis, siponimod, multiple sclerosis, population pharmacokinetics, noncompartmental analysis

## Abstract

**Objectives:** This study aimed to investigate the pharmacokinetic characteristics of siponimod in healthy volunteers and patients with MS based on aggregated data from published clinical trials, and to explore the factors influencing siponimod exposure.

**Methods:** A total of 476 siponimod plasma concentrations aggregated from 28 dosage groups (corresponding to 294 healthy volunteers and 207 patients with MS) were collected from published clinical trials. Population pharmacokinetic (PPK) analysis was performed using a nonlinear, mixed-effect modeling approach. The pharmacokinetic properties of siponimod in healthy volunteers and patients with MS were compared, and the influence of covariates on siponimod exposure was evaluated using both PPK analysis and noncompartmental analysis (NCA).

**Results:** A one-compartment model with first-order absorption and elimination adequately described siponimod pharmacokinetics. The typical population parameter estimates of clearance (CL/F), apparent volume of distribution (V/F), and absorption rate constant (ka) were 3.17 L/h, 112.70 L, and 0.38 h^−1^, respectively. An 11.85% lower siponimod clearance was estimated for patients with MS relative to healthy volunteers. Subgroup analyses using NCA assessments revealed that siponimod presented an accumulation index of approximately 2 after multiple administration. Compared with nonobese participants, obese participants had a relatively lower dose-corrected area under the concentration-time curve (AUC_0-∞_/D) (0.31 vs. 0.42 h/L) and V/F (120.95 vs. 133.75 L), and a relatively higher CL/F (3.25 vs. 3.21 L/h). Participants with CYP2C9*2/*3, *1/*3, and *3/*3 genotypes experienced an increased (1.3- and 3.4-fold, respectively) AUC_0-∞_/D and a decreased (0.7- and 0.3-fold, respectively) CL/F compared with those in participants with the CYP2C9*1/*1, *1*2, and *2*2 genotypes. Fluconazole combination led to a decrease in CL/F (approximately 0.5 times) and an increase in AUC_0-∞_/D (approximately 1.3 times).

**Conclusion:** Siponimod pharmacokinetic properties in healthy volunteers and patients with MS were explored using complementary model-based meta-analysis (MBMA) and NCA approaches. A slightly lower siponimod clearance was observed in patients with MS than in healthy volunteers. The dosage regimen, body mass index, CYP2C9 genetic polymorphism and fluconazole combination may had influences on siponimod pharmacokinetics. Such model paves the road to more population-based analyses in different patient populations with MS to quantify the effect of any influencing factors on siponimod pharmacokinetics.

## 1 Introduction

Multiple sclerosis (MS) is an immune-mediated disorder influencing the central nervous system (CNS), causing demyelination related to axonal and neuronal degeneration (Reich et al., 2018), eventually resulting in increased disability. About 85% of the patients have a relapsing-remitting disease course first, typically converted into a secondary progressive phase (SPMS), characterized by progressive disability accrual, with or without superimposed relapses (attacks). For the other patients, the clinical classification is primary progressive (Weinshenker et al., 1989; Lublin and Reingold, 1996). MS is classified as a rare disease in China with a national incidence of 0.055 per 100,000 in children and 0.288 per 100,000 in adults (Tian et al., 2020).

Despite no definite cure for MS, several disease-modifying treatments (DMTs) have become available to reduce the risk of recurrence and disability progression (Gholamzad et al., 2019). These include siponimod, a new-generation sphingosine-1-phosphate (S1P) receptor modulator with specificity for the S1P1 and S1P5 subtypes (Gergely et al., 2012). Compared with the first oral DMT fingolimod, siponimod has the advantages of a lower risk of bradycardia and a shorter washout time, with the restoration of lymphocyte counts within a week. The results from EXPAND trial demonstrated that siponimod was modestly effective in SPMS compared with placebo, with 21% and 26% risk reductions for the 3- and 6-month confirmed EDSS progression (Kappos et al., 2018; Dumitrescu et al., 2019). Siponimod was approved for marketing by the Food and Drug Administration (FDA) in March 2019, and was approved successively by Europe, China, and Japan in 2020 due to its outstanding performance in clinical trials, and thus has become the first oral therapeutic drug in the world for patients with SPMS.

Studies in healthy subjects revealed that following oral intake, siponimod undergoes slow-to-medium but almost complete intestinal absorption, achieving the maximum plasma concentration in about 4 h. The mean volume of distribution (Vd) of siponimod is 124 L. It is metabolized extensively, mainly by CYP2C9 (79.3%) and CYP3A4 (18.5%). The main metabolites, M3 and M17, are considered not to affect the efficacy and safety of siponimod. Siponimod presents a linear elimination within the dose range of 0.1–75 mg, with a half-life of about 30 h and an estimated complete washout of about 6.3–7 days. Siponimod is eliminated mainly *via* metabolism and subsequent biliary/fecal excretion (Gergely et al., 2012; Glaenzel et al., 2018; Jin et al., 2018; Scott, 2020).

Comprehensive assessments such as CYP2C9 genotype determination, complete blood count, and ophthalmic and cardiac evaluations are needed prior to the first dose of siponimod. The dosage regimen according to the CYP2C9 genotype is required, and the initial titration is recommended. In patients with CYP2C9*1/*1, *1/*2 or *2/*2 genotypes, dose titration starts with 0.25 mg once daily on days 1 and 2, followed by 0.5 mg on day 3, 0.75 mg on day 4, and 1.25 mg on day 5, to reach the maintenance dose of 2 mg once daily starting on day 6. For patients with CYP2C9*1/*3 or *2/*3 genotypes, the titration period is reduced to 4 days, that is, 0.25 mg once daily on days 1 and 2, 0.5 mg on day 3 and 0.75 mg on day 4, followed by a maintenance dose of 1 mg once daily starting on day 5 (Novartis Pharamceuticals Corporation, 2022). Due to the particularity of siponimod administration, pharmacokinetic characteristic of siponimod in different populations needs to be fully considered. Since inter-trial differences often occur between pharmacokinetic studies, this study aimed to use the model-based meta-analysis (MBMA) method to summarize the pharmacokinetic data of existing studies, to conduct a comprehensive evaluation of the pharmacokinetic characteristics of siponimod, and to obtain more representative pharmacokinetic parameters. Namely, to propose a PPK model of siponimod in healthy participants and patients with MS, to generate new estimates at the population level by integrating the pooled data from multiple dosage groups, and further to quantitatively evaluate the impact of potential factors on siponimod pharmacokinetics through the pooled analysis of multiple trials, thus to provide a reference for its precise medication.

## 2 Materials and Methods

### 2.1 Literature Search and Data Extraction

A systematic literature search of PubMed, Embase, the Cochrane Library, and ClinicalTrials databases from inception to March 2021 was carried out to identify published clinical pharmacokinetic studies on siponimod. The keywords used for the search were “siponimod,” “Mayzent,” “BAF312,” and “BAF-312.” The search was restricted to studies that included healthy participants or patients with MS taking siponimod orally and reported in the English language. The initial search resulted in 31 studies. After full-paper examination, studies that had the mean plasma concentration–time profiles tabulated or plotted were included. Additional data including, but not limited to, grouping information and sample size of each group, demographics [race, sex, age, weight, height, body mass index (BMI)], siponimod administration information (administration time, dosage amount and interval), laboratory test results (lymphocyte count, heart rate, creatinine clearance, and eGFR), CYP2C9 genetic polymorphism, and fluconazole co-administration were also extracted in the dataset for analysis. The aforementioned information was extracted independently by two reviewers, and the inconsistencies were determined through consultation with the third reviewer. Aggregate (mean) plasma concentration-time data of identified publications were extracted by GetData Graph digitizer software (version 2.20). The data extraction error of the two reviewers when reading the graphs was controlled within 2%. If the error exceeded 2%, the data was extracted again, and the average of the extracted values was used as the final data for analysis.

### 2.2 Population Pharmacokinetic Model Development

#### 2.2.1 Structural PK Model

Nonlinear mixed-effects modeling was completed using NONMEM 7.4.3 (ICON Development Solutions, MD, United States), Perl Speaks NONMEM (PsN) 3.2.12 (Uppsala University, Sweden), and R 3.5.1 software. The first-order conditional estimation method with interactions option with η-ε interaction was adopted, and the ADVAN2 TRANS2 subroutine was selected.

A graphical exploration of plasma concentration–time data were conducted to assess siponimod pharmacokinetic characteristics, possible influencing factors, and outliers. The PPK model was established in a stepwise manner. Combined with the graphical exploration results, the basic structural model was selected according to the pharmacokinetic characteristics of siponimod. One-, two- and three-compartment models with additive, proportional and mixed error were tested respectively, and the basic model was selected by comparing their objective function value (OFV) as well as the complexity of the models. Inter-study variability (ISV) was added using the following exponential error model (Klünder et al., 2018):

$$P_{ni}=\theta_{n}\cdot \exp\;\left(\eta_{ni}\right)$$ (1)

where *P*_*ni*_, *θ*_*n*_, and *η*_*ni*_ represented the individual study estimated parameter, population estimated parameter, and inter-study random effect for *i*_th_ study and *n*_th_ parameter, respectively. The *η*_*n*_ were assumed to be normally distributed with a mean of 0 and a variance of *ω*_*n*_^2^.

The residual variability (RV) was described as a combination of proportional and additive error terms (Klünder et al., 2018):

$$C_{ij}={\hat{C}}_{ij}\cdot \left(1+\varepsilon_{1ij}\right)+\varepsilon_{2ij}$$ (2)

where *C*_*ij*_ and *Ĉ*_*ij*_ are, respectively, the observed and predicted plasma concentrations for study *i* at time *j*, and *ε*_*1ij*_ and *ε*_*2ij*_ are, respectively, the proportional and additional residuals of the measured concentrations, which were independently distributed normally with a mean of 0 and variances of *σ* ^2^.

#### 2.2.2 Covariate Analysis

When performing covariate analysis for continuous covariates, average values (arithmetic means) were preferred over median values for analysis since most studies reported the average values of the covariates. For covariates with missing average values, median values were extracted instead. For covariates reported in the form of a range, the mean value of the range was assigned. For covariates with neither average nor median values (namely, missing covariates), if the missing ratio was no greater than 50% of the total covariate data in the MBMA database, weighted average values according to the sample sizes of dosage groups were imputed for continuous covariates, and the most common category of the MBMA dataset was imputed for categorical covariates. If the missing ratio was greater than 50% of total covariate data in the MBMA database, the covariates were not included in the analysis.

Covariate analysis was performed using a forward inclusion/backward elimination process. The likelihood ratio test was used to test the effect of each variable on model parameters. The effects of sex, race, age, BMI, study population (healthy participants or patients with MS), and fluconazole co-administration were investigated as potential covariates. Continuous covariates were assessed using the following equation:

$$\theta_{i}=\theta_{T}\cdot \exp\left(k_{Cov}\cdot \ln\left(\frac{\mathrm{Co}v_{i}}{\mathrm{Co}v_{\mathrm{pop}}}\right)+\eta_{i}\right)$$ (3)

Categorical covariates were assessed using the following equation:

$$\theta_{i}=\theta_{T}\cdot \exp\left(k_{Cov}\cdot X_{i}+\eta_{i}\right)$$ (4)

where *θ*_*i*_ is the individual study parameter for study *i*; *θ*_*T*_ is the corresponding typical value of the parameter; *Cov*_*i*_ is the individual study value of the continuous covariate in study *i*, *Cov*_*pop*_ is the population median value of the continuous covariate; *X*_*i*_ is the individual study categorical covariate indicator, with 0 representing the most frequent category and other integer values representing other categories; *k*_*cov*_ is the coefficient representing the strength of the covariate effect; and *η*_*i*_ is the inter-study random effect.

Selected covariates were incorporated into the structural model one by one during the forward inclusion procedure, and covariates with a significant decrease (reduction >3.84, *p* < 0.05, *χ* ^2^ distribution with one degree of freedom) in the OFV from the structural model were included in the full regression model. Then, each covariate was removed from the full regression model independently. Covariates resulting in an increase in OFV greater than 6.63 (*p* < 0.01, *χ* ^2^ distribution) were retained in the final model.

### 2.3 Model Evaluation

The goodness-of-fit assessment was performed by plotting the observed concentrations against the corresponding individual (IPRED) and population predictive concentrations (PRED) as well as the conditional weighted residual errors (CWRES) against PRED and time. The stability and reliability of the PPK model were evaluated by the bootstrap resampling method. Further, 1,000 times resampling was completed, and the values of estimated parameters and their 95% confidence intervals (CIs) (2.5th percentile and 97.5th percentile) from the bootstrap procedure were compared with those from the original dataset. pcVPCs were performed based on 1,000 simulations to evaluate the appropriateness of the PPK model graphically. Furthermore, the shrinkage extent on each parameter (η-shrinkage) and on individual predictions (ε-shrinkage) was assessed.

### 2.4 Analysis of Pharmacokinetic Influencing Factors

The PPK and NCA approaches were performed separately, and the investigation of factors affecting siponimod pharmacokinetics was conducted by combing the results of the two approaches. CL/F and V/F values of individual dosage groups were predicted by the final PPK model, additionally, the dose-corrected area under the concentration–time curve (AUC_0-∞_/D), dose-corrected peak concentration (*C*_max_/D), CL/F, and V/F were derived from plasma concentration–time profiles using NCA (Phoenix WinNonlin Version 8.2.0.4383). The impacts of study population (healthy volunteers or MS patients), dosage regimen (single or multiple doses), BMI (BMI <28, BMI ≥28 kg/m^2^), CYP2C9 genotype (CYP2C9*1/*1 or *1/*2 or *2/*2, CYP2C9*2/*3 or *1/*3, CYP2C9*3/*3), and fluconazole combination on the pharmacokinetics of siponimod were assessed by categorizing the participants into different subgroups based on the aforementioned variables and grouping criteria. To investigate the trends of the aforementioned variables among subgroups, the weighted geometric means and weighted geometric coefficient of variations in the pharmacokinetic parameters of the study population subgroups were calculated and compared according to PPK prediction results, and that of the other subgroups were calculated and compared according to NCA estimates. When calculating the weighted geometric mean and the weighted geometric coefficient of variation, the weighting was based on the sample sizes of the dosage groups.

## 3 Results

### 3.1 Data Summary and Demographics

The PPK analysis was conducted using combined data from 10 studies conducted in healthy participants and one study conducted in patients with MS (Table 1). Although siponimod intravenous infusion was mentioned in one study (Shakeri-Nejad et al., 2020), only data for the oral administration of siponimod was included in the analysis. Siponimod was administered either by single-dose regimens (0.25–10 mg) or by multi-dose regimens, including dose escalation (0.25–20 mg once daily) and titration followed by maintenance dose (0.5–10 mg once daily). In terms of blood sample collection, most studies adopted dense sampling strategies, while the others used sparse sampling data. Siponimod was combined with rifampicin, itraconazole, and fluconazole in three studies. Studies with no washout period between monotherapy and combination therapy were excluded, and only the data of fluconazole combination was retained (Gardin et al., 2019b).

**TABLE 1:** Overview of studies included in the siponimod PPK analysis.

Study	Population	Number of participants enrolled	Study design	Siponimod dose (mg)	Pharmacokinetic sampling	Concomitant medication	References
1	Healthy participants	48	Multiple-dose, randomized, placebo-controlled	Cohort 1: 0.3 mg p.o. qd for 28 days	Days 1, 2, 4, 6, 7, 8, 11, 14, 17, 20, 23, 26, 28, 29, 30, 32, 35, 38, 42, and 49	—	Gergely et al. (2012)
				Cohort 2: 1 mg p.o. qd for 28 days			
				Cohort 3: 2.5 mg p.o. qd for 28 days			
				Cohort 4: 10 mg p.o. qd for 28 days			
				Cohort 5: 20 mg p.o. qd for 28 days			
2	Healthy participants	56	Multiple-dose, randomized, placebo-controlled	Group 1: 0.5 mg p.o. on day 3, 0.75 mg p.o. on day 4, 1 mg p.o. on day 5, 2 mg p.o. on day 6, 4 mg p.o. on day 7, 8 mg p.o. on day 8, and 10 mg p.o. qd on days 9–12	Pre-dose; 2, 3, 4, 6, 8, and 12 h post-dose throughout the study; and 24 h post-dose on day 12	—	Legangneux et al. (2013)
				Group 2: 0.25 p.o. qd on days 1–2, 0.5 p.o. on day 3, 0.75 p.o. on day 4, 1.25 mg p.o. on day 5, 2 mg p.o. on day 6, 3 mg p.o. on day 7, 5 mg p.o. on day 8, 8 mg p.o. on day 9, and 10 mg p.o. qd on days 10–12			
				Group 3: 10 mg p.o. qd on days 1–12			
3	Healthy participants	304	Multiple-dose, randomized, placebo- and moxifloxacin- controlled	0.25 mg p.o. qd on days 1–2, 0.5 mg p.o. on day 3, 0.75 mg p.o. on day 4, 1.25 mg p.o. on day 5, 2 mg p.o. qd on days 6–10, 3 mg p.o. on day 11, 5 mg p.o. on day 12, 8 mg p.o. on day 13, and 10 mg p.o. qd on days 14–18	Pre-dose on day –1; pre-dose; 0.5, 1, 2, 3, 4, 6, and 24 h post-dose on days 10 and 18	—	Shakeri-Nejad et al. (2015)
4	Renal-impaired participants and healthy participants	16	Single-dose, nonrandomized, parallel-group	0.25 mg p.o. once	Pre-dose; 0.25, 0.5, 0.75, 1, 1.5, 2, 3, 4, 6, 8, 12, 16, 24, 36, 48, 72, 96, 144, 216, and 312 ± 24 h post-dose	—	Gardin et al. (2017)
5	Hepatic-impaired participants and healthy participants	40	Single-dose, nonrandomized, parallel-group	0.25 mg p.o. once	Pre-dose; 0.25, 0.5, 0.75, 1, 1.5, 2, 3, 4, 6, 8, 12, 16, 24, 36, 48, 72, 96, 144, 216, 312, 408, and 504 h post-dose	—	Shakeri-Nejad et al. (2017)
6	Healthy participants	4	Single-dose, nonrandomized, noncontrol	10 mg p.o. once	Pre-dose; 1, 2, 4, 6, 8, 12, 16, 24, 36, 48, 72, 96, 120, 144, 168, 192, 216, 240, 312, 480, and 816 h post-dose	—	Glaenzel et al. (2018)
7	Healthy participants	16	Multiple-dose, nonrandomized, noncontrol	Period 1: 0.25 mg p.o. qd on days 1–2, 0.5 mg p.o. on day 3, 0.75 mg p.o. on day 4, 1.25 mg p.o. on day 5, and 2 mg p.o. qd on days 6–12	Pre-dose on days 1, 3, 4, 5, 8, 10, 12, 13, 15, 17, 19, 21, 22, 23, 24, and 25	Rifampin	Gardin et al. (2018)
				Period 2: 2 mg p.o. qd on days 13–24	4 h post-dose on days 12 and 24		
8	Healthy participants	30	Single-sequence, nonrandomized, parallel-group	0.25 mg p.o. once on days 1 (period 1) and 19 (period 3)	Period 1: Pre-dose; 0.25, 0.5, 1, 2, 3, 4, 6, 8, 12, 24, 36, 72, 96, 144, 192, 240, 312	Itraconazole	Gardin et al. (2019a)
					Period 3: postdose at 0.25, 0.5, 1, 2, 3, 4, 6, 8, 12, 24, 36, 72, 96, 120, 144, 168, 216, 264, and 312 h		
9	Healthy participants	Study A: 14	Study A: single-dose, nonrandomized, noncontrol	Study A: 4 mg p.o. once on days 1 (Period 1) and 3 (Period 2)	Study A: pre-dose; 0.25, 0.5, 0.75, 1, 1.5, 2, 3, 4, 6, 8, 12, 16, 24, 36, 48, 72, 96, 144, 216, 312 (Period 1 and 2), and 408 h post-dose (Period 2)	Fluconazole	Gardin et al. (2019b)
		Study B: 24	Study B: multiple-dose, nonrandomized, noncontrol	Study B: 0.25 mg p.o. once on day 1 (Part 1), 0.25 mg p.o. qd on days 1 and 2, and 0.5 mg p.o. once on day 3 (Part 2)	Study B: Part 1: pre-dose; 0.25, 0.5, 0.75, 1, 1.5, 2, 3, 4, 6, 8, 12, 16, 24, 36, 48, 72, 96, 144, 216, 312, 408, 504, 600, 720, 840, and 984 h post-dose		
					Part 2: predose on days 1–3, and 0.25, 0.5, 0.75, 1, 1.5, 2, 3, 4, 6, 8, 12, 16, and 24 h post-dose on day 3		
10	Healthy participants	Part 1: 16	Part 1: Two-sequence, randomized, cross-over	Part 1: 0.25 mg p.o. once followed by 0.25 mg i.v.gtt once (sequence 1) or 0.25 mg i.v.gtt once followed by 0.25 mg p.o. once (sequence 2)	Part 1: pre-dose; 0.25, 0.5, 0.75, 1, 1.5, 2, 3, 4, 6, 8, 12, 24, 36, 48, 72, 96, 144, 216, 312, and 336 h post-dose from day 1 and day 15	—	Shakeri-Nejad et al. (2020)
		Part 2: 17	Part 2: single-dose, nonrandomized, noncontrol	Part 2: 1 mg i.v.gtt once	Part 2: predose, 0.25, 0.5, 0.75, 1, 2, 4, 6, 8, 12, 16, 18, 24, 48, 72, 96, 144, 216, 312, and 336 h post-dose from day 1		
11	Patients with MS	297	Dose-ranging, randomized, placebo-controlled	Cohort 1: 10 mg (group 1), 2 mg (group 2), and 0.5 mg (group 3) p.o. qd for 6 months	Pre-dose on days 30, 90, and 180	—	Selmaj et al. (2013)
				Cohort 2: 1.25 mg (group 1) and 0.25 mg (group 2) p.o. qd for 3 months			

p.o., Oral administration; i.v.gtt, intravenous administration; q.d., once-daily.

A total of 476 concentrations from 28 dosage groups (aggregated data from 294 healthy participants and 207 patients with MS) were included in the PPK analysis (Table 2). According to available data, studies in healthy participants mainly enrolled male volunteers (88.8%), while studies in patients with MS mostly included women (71.0%). The majority of participants included were Caucasians (85.4%). Four studies reported the CYP2C9 genotypes of the included participants. The participants with CYP2C9*1/*1, CYP2C9*1/*2, CYP2C9*1/*3, CYP2C9*2/*3, and CYP2C9*3/*3 accounted for 52.3%, 19.3%, 14.8%, 6.8%, and 6.8%, respectively. Fluconazole was used in combination with siponimod in 14 participants.

**TABLE 2:** Baseline characteristics for participants included in the PPK analysis data set.

Characteristics	Study 1	Study 2 (1 discontinued)	Study 3	Study 4	Study 5	Study 6	Study 7	Study 8 (1 discontinued)	Study 9	Study 10 (1 discontinued)	Study 11 (28 discontinued)
Participants [*n* (%)]											
Healthy participants	37 (100.0)	42 (100.0)	92 (100.0)	8 (100.0)	14 (100.0)	4 (100.0)	16 (100.0)	30 (100.0)	38 (100.0)	16 (100.0)	0 (0.0)
Patients with MS	0 (0.0)	0 (0.0)	0 (0.0)	0 (0.0)	0 (0.0)	0 (0.0)	0 (0.0)	0 (0.0)	0 (0.0)	0 (0.0)	235 (100.0)
Sex [*n* (%)]											
Male	NR	39 (92.9)	82 (89.0)	4 (50.0)	9 (64.3)	4 (100.0)	15 (93.8)	28 (93.3)	35 (92.1)	15 (93.8)	68 (28.9)
Female		3 (7.1)	10 (10.9)	4 (50.0)	5 (35.7)	0 (0.0)	1 (6.3)	2 (6.7)	3 (7.9)	1 (6.3)	167 (71.0)
Race [*n* (%)]											
Caucasian	NR	40 (95.2)	81 (88.0)	6 (75.0)	14 (100.0)	4 (100.0)	6 (37.5)	27 (90.0)	34 (89.5)	10 (62.5)	NR
Others		2 (4.8)	11 (12.0)	2 (25.0)	0 (0.0)	0 (0.0)	10 (62.5)	3 (10.0)	4 (10.5)	6 (37.5)	
Age, year (mean ± SD), [median (min–max)]	18.0–55.0	36.2 ± 7.7	35.0 ± 7.6	54.9 ± 5.3	50.1 ± 5.0	35.8 (18.0–54.0)	31.1 ± 8.3	36.7 ± 8.7	35.3 ± 15.8	32.9 ± 7.5	36.6 ± 8.6
BMI, kg/m^2^ (mean ± SD)	NR	NR	26.1 ± 2.6	29.5 ± 3.8	27.1 ± 3.8	NR	25.8 ± 3.0	25.4 ± 2.6	24.0 ± 2.5	26.3 ± 2.4	NR
CYP2C9 genotype [*n* (%)]											
CYP2C9*1*1	NR	NR	NR	NR	NR	4 (100.0)	NR	0 (0.0)	26 (68.4)	16 (100.0)	NR
CYP2C9*1*2						0 (0.0)		17 (56.7)	0 (0.0)	0 (0.0)	
CYP2C9*1*3						0 (0.0)		13 (43.3)	0 (0.0)	0 (0.0)	
CYP2C9*2*3						0 (0.0)		0 (0.0)	6 (15.8)	0 (0.0)	
CYP2C9*3*3						0 (0.0)		0 (0.0)	6 (15.8)	0 (0.0)	
Fluconazole combination [*n* (%)]	0 (0.0)	0 (0.0)	0 (0.0)	0 (0.0)	0 (0.0)	0 (0.0)	0 (0.0)	0 (0.0)	14 (36.8)	0 (0.0)	0 (0.0)

NR, not reported.

### 3.2 Population Pharmacokinetic Analysis

By comparing the one-, two- and three-compartment models, the one-compartment model with mixed error was selected as the basic model owing to its lowest OFV and conciseness (Supplementary Table S1). During forward inclusion, BMI and fluconazole combination were incorporated into the full regression model with a significant drop in the OFV of 5.52 and 5.84 units, respectively, but they were both removed during the backward elimination procedure. Other covariates, such as age, sex, race and study population, showed no significant effects on the CL/F and V/F of siponimod. The final model had the following structure:

$$\mathrm{CL}/F\left(L/h\right)=TVCL\times \exp\left(\eta_{CL}\right)$$

$$V/F\left(L\right)=TVV\times \exp\left(\eta_{V}\right)$$

$$Ka\left(h^{\mathrm{-1}}\right)=TVKa\times \exp\left(\eta_{Ka}\right)$$ (5)

The final estimated siponimod CL/F was 3.17 L/h, V/F was 112.70 L, and Ka was 0.38 h^−1^. The PPK parameters of the final model are presented in Table 3.

**TABLE 3:** Parameter estimates, standard error, and bootstrap confidence intervals of the pharmacokinetic final model.

Pharmacokinetic parameters	Estimates (RSE%)	738 successful bootstrap median (95% CI)
PK parameter		
CL/F (L/h)	3.17 (5.80)	3.19 (2.83–3.51)
V/F (L)	112.70 (5.20)	113.00 (102.00–125.00)
Ka (h^−1^)	0.38 (9.10)	0.38 (0.31–0.44)
ISV		
CL/F (%)	28.93 (27.95)	27.20 (7.34–43.00)
V/F (%)	23.29 (28.25)	22.00 (5.80–32.60)
Ka (%)	22.48 (47.60)	24.30 (6.16–38.90)
RV		
Proportional error (%)	24.40 (21.00)	23.40 (10.70–33.10)
Additive error (ng/ml)	0.13 (22.20)	0.13 (0.09–0.22)

CI, confidence interval; ISV, inter-study variability; PK, pharmacokinetic; RSE, relative standard error; RV, residual variability.

### 3.3 Model Evaluation

**FIGURE 1:**
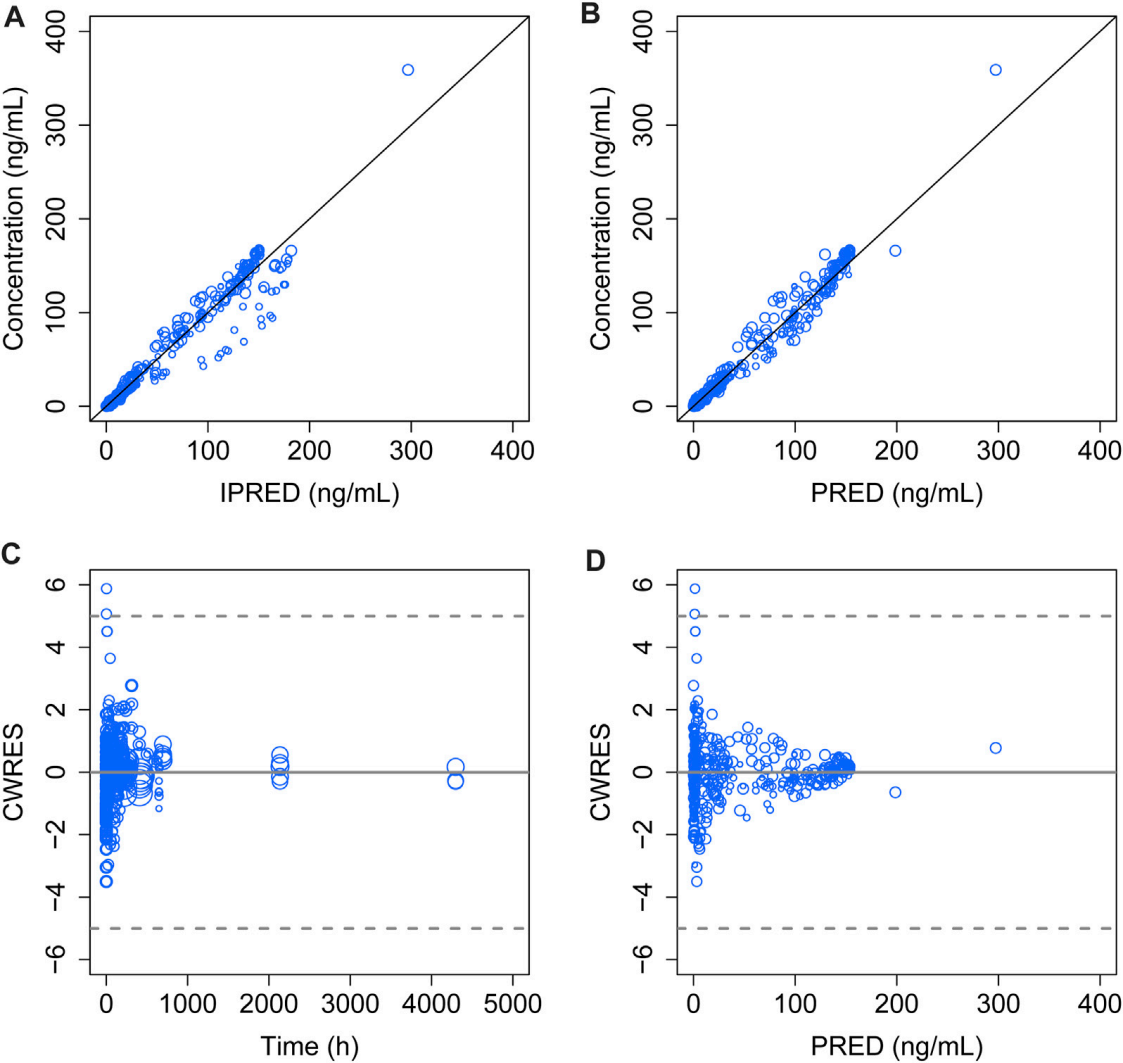
Goodness-of-fit plot of the final model. **(A)** Observed concentrations versus IPERD. **(B)** Observed concentrations versus PRED. **(C)** CWRES versus time. **(D)** CWRES versus PRED. The black lines in **(A)** and **(B)** represent the regression line, and the gray solid lines in **(C)** and **(D)** indicate the position where CWRES equals zero. The size of the open circles corresponds to the sample size of each dosage group.

**FIGURE 2:**
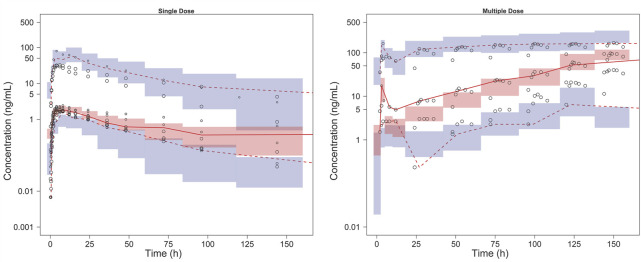
pcVPC results of the final model. The solid line and dashed lines, respectively, represent the median and 95% CI of the observations. Open circles represent the observed concentrations, the size of which corresponds to the sample size of each dosage group. The shaded red area represents the 95% CI of medians, and the shaded blue areas stand for the 95% CI of the 2.5th and 97.5th percentiles of the simulation results.

The model diagnostic plots demonstrated acceptable goodness-of-fit for the final model. Most values in the plot of PRED and IPRED versus observed concentrations were close to the identity line, revealing that the model adequately described most of the observed siponimod concentrations. No systematic trends were observed in the diagnostic plots of CWRES versus time and PRED, indicating that the model was reasonably unbiased (Figure 1). pcVPC results for siponimod single and multiple doses confirmed an adequate predictive power of the final model, with the observed data included in the range of 95% CI; the median and 95% CI lines of the observations were located near the middle of the 1,000 simulation results (Figure 2). Partly limited by the study sample size and data sources, a total of 738 bootstrap runs converged successfully. The estimated pharmacokinetic parameter values from the original data set were close to the median values estimated from the bootstrap verification, indicating a relatively good stability of the final model (Table 3). The estimates of the shrinkage were 5.25%, 23.66%, and 44.11% for ETAs of CL/F, V/F, and ka, respectively, and were both 4.79% for additive and proportional residual errors.

### 3.4 Analysis of Pharmacokinetic Influencing Factors

The covariate screening during the PPK modeling process did not filter out any significant covariates. When calculated using the final PPK model, an 11.85% lower CL/F was estimated for patients with MS relative to healthy volunteers, while their V/F did not show too much difference (Table 4).

**TABLE 4:** PPK and NCA pharmacokinetic parameter estimates and subgroup analysis.

Subgroup	Participants^a^, *n* (%)	Weighted geometric mean (weighted geometric coefficient of variation, %)			
		AUC_0-∞_/D (h/L)	*C*_max_/D (L^−1^)	CL/F (L/h)	V/F (L)
Study population^b^					
Healthy volunteers	318 (60.57)	NA	NA	3.39 (24.21)	109.85 (18.44)
MS patients	207 (39.43)	NA	NA	2.98 (4.83)	113.58 (0.64)
Dosage regimen^c^					
Single dose	132 (49.81)	0.34 (38.75)	0.0076 (10.04)	2.97 (38.75)	143.27 (14.37)
Multiple doses	133 (50.19)	0.57 (5.44)	0.0155 (3.14)	3.52 (5.10)	116.61 (8.92)
BMI (kg/m^2^)^c^					
BMI < 28	212 (96.36)	0.42 (40.07)	0.0103 (37.54)	3.21 (31.85)	133.75 (10.13)
BMI ≥ 28	8 (3.64)	0.31 (0.00)	0.0087 (0.00)	3.25 (0.00)	120.95 (0.00)
CYP2C9 genotype^c^					
CYP2C9*1/*1 or *1/*2 or *2/*2^d^	75 (75.00)	0.32 (27.72)	0.0075 (7.13)	3.11 (27.72)	148.17 (15.56)
CYP2C9*2/*3 or *1/*3	19 (19.00)	0.43 (19.32)	0.0077 (15.09)	2.31 (19.32)	140.56 (4.25)
CYP2C9*3/*3	6 (6.00)	1.10 (0.00)	0.0092 (0.00)	0.91 (0.00)	181.36 (0.00)
Fluconazole combination^c^					
Yes	14 (5.28)	0.56 (0.00)	0.0081 (0.00)	1.78 (0.00)	165.15 (0.00)
No	251 (94.72)	0.43 (39.11)	0.0110 (38.01)	3.34 (25.14)	127.45 (15.11)

NA, not applicable.

a Analysis based on unfilled raw data; If a subject was in different periods, he/she was counted as different individuals.

b Calculated according to PPK prediction results.

c Calculated according to NCA estimates.

d Pharmacokinetic parameter estimates were similar between participants with CYP2C9*1/*1 and participants with CYP2C9*1/*2 or *2/*2, and therefore the aforementioned populations were integrated into one group.

The NCA analysis included eight studies after excluding studies that did not implement an intensive sampling strategy (Gergely et al., 2012; Selmaj et al., 2013; Gardin et al., 2018). Pharmacokinetic parameter estimates and subgroup analysis based on NCA revealed that the weighted geometric mean of AUC_0-∞_/D for patients who received multiple doses of siponimod was 1.67 times that of patients receiving single doses, and *C*_max_/*D* was 2.04 times that of participants receiving single doses. Compared with participants whose BMI was <28 kg/m^2^, a slight decrease in AUC_0-∞_/D (0.31 vs. 0.42 h/L) and a slight increase in CL/F (3.25 vs. 3.21 L/h) were observed in a population with BMI ≥28 kg/m^2^.

In terms of CYP2C9 genetic polymorphism, the weighted geometric mean of AUC_0-∞_/D among the three subgroups (group 1: CYP2C9*1/*1 or *1/*2 or *2/*2 subgroup, group 2: CYP2C9*2/*3 or *1/*3 subgroup, group 3: CYP2C9*3/*3 subgroup) showed an obvious increasing trend (1.3 and 3.4 times, respectively, that of group 1), while CL/F of the aforementioned three subgroups presented an apparent decreasing trend (0.7 and 0.3 times, respectively, that of group 1). The combination of fluconazole led to a decrease in CL/F (approximately 0.5 times) and an increase in AUC_0-∞_/D (approximately 1.3 times) (Table 4).

## 4 Discussion

MS is a considerable socioeconomic burden because it reflects the most common cause of nontraumatic neurological disability among young adults (Rush et al., 2015). It affects more than 2.3 million people worldwide (Thompson et al., 2018). Nearly one million patients were confirmed with MS, with associated costs of more than $24 billion per year in the United States (Wallin et al., 2019). In the last decades, significant progress has been made in the treatment of MS with the successive approval of DMT drugs.

Siponimod is a functional antagonist that results in significant and long-term S1P1 internalization (Gergely et al., 2012; Pan et al., 2013). Its main action mechanism on MS is the depletion of circulating lymphocytes, thus preventing CNS infiltration. It easily penetrates the blood–brain barrier, and hence may facilitate CNS repair directly by mediating S1P1 on astrocytes and S1P5 on oligodendrocytes (Gergely et al., 2012). Though several traditional siponimod pharmacokinetic studies and two physiologically based pharmacokinetic (PBPK) models (Jin et al., 2018; Huth et al., 2019) have been published, the factors affecting siponimod pharmacokinetics have not been thoroughly explored.

This study proposed a siponimod PPK model using the MBMA method. MBMA allowed increasing the sample size by integrating the mean plasma concentration–time data of siponimod at various dosage regimens after oral administration of single and multiple doses. As shown in Table 3, siponimod pharmacokinetics was well characterized, and the model parameters were well estimated with high accuracy (RSE ≤10%). The typical population value of CL/F and V/F of the final model was 3.17 L/h and 112.70 L, respectively, which were consistent with the data of previous literature reports (CL = 3.120 L/h; V = 2.12 L/kg) (Jin et al., 2018; Huth et al., 2019). A good stability and accuracy of the final model were confirmed by goodness-of-fit, bootstrap, VPC, and shrinkage estimates.

Since the included studies reported only the means or medians of the covariates, they were information-poor. Aggregated data has a certain disadvantages in establishing covariate models, namely: the small ranges of covariates at the mean level, incapability to use the summary level covariates to extrapolate to the patient level because of the ecological bias, and the small numbers of studies involved in MBMA (Boucher and Bennetts, 2018). Taking into account the above limitations, dual approaches were applied to yield as much information as possible: covariate analysis in the PPK framework and subgroup analysis based on the pharmacokinetic parameters calculated using PPK and NCA. NCA analysis revealed that dosage regimen, BMI, CYP2C9 genetic polymorphism and fluconazole combination might affect siponimod pharmacokinetics, among which BMI and fluconazole combination also showed an influence on siponimod pharmacokinetic parameters during the forward inclusion process in the PPK analysis, though the effect was not significant enough to be retained in the final model possibily due to the aggregrated data. Other covariate, such as CYP2C9 genotype, could not be examined in the PPK process due to its high missing ratio, therfore it was investigated by NCA analysis, and obtained relatively obvious results, which were consistent with clinical experience. Therefore, for pharmacokinetic study based on summary data, perhaps NCA may serve as a complement to the PPK approach. The subgroup analyses based on NCA revealed the trend of variables among different subgroups, providing a basis for the preliminary exploration of siponimod pharmacokinetic properties and the design of subsequent pharmacokinetic and PPK studies.

NCA assessments revealed that after multiple dosing, the weighted geometric mean of *C*_max_/D of siponimod demonstrated an accumulation index of approximately two compared with single doses, consistent with the accumulation ratio of 1.9–2.7 observed by Gergely et al. (2012).

BMI, calculated by dividing the total bodyweight (kg) by the square of the height (m), is the World Health Organization’s preferred measurement for obesity. BMI subgroups were divided according to the Cooperative Meta-analysis Group of China Obesity Task Force report, which recommended BMI at 28 mg/m^2^ as the cut-off point for obesity (Zhou, 2002). As shown in Table 4, obese participants tended to present a relatively higher CL/F and a lower AUC_0-∞_/D and V/F compared with other participants. The alterations in siponimod V/F might be related to drug properties such as ionization properties, lipophilicity, blood: plasma ratio, and protein binding, which were difficult to predict. For hepatically metabolized drugs such as siponimod, forecasting drug clearance in obesity is also challenging. Besides CYP enzyme activity, factors such as liver size, drug extraction ratio, duration of obesity, and influence of transporters should also be considered (Smit et al., 2018). A previous study confirmed that weight was one of the covariates that affected the pharmacokinetics of siponimod (Gardin et al., 2019b). However, there is currently no evidence that within the normal weight range, the impact of weight alone is clinically significant, thus no dose adjustment is considered warranted. BMI was found to have an impact on the pharmacokinetics of siponimodin this study, though the clinical significance of which needs further verification.

Siponimod is mostly eliminated via oxidative metabolism with CYP2C9 as the predominant hepatic enzyme (Jin et al., 2018). A previous study showed that participants with CYP2C9*2/*3 and CYP2C9*3/*3 genotypes demonstrated an increased (twofold to fourfold) AUC compared with participants with CYP2C9*1/*1 genotype (Goodman et al., 2019). NCA in this study showed comparable results. Participants with CYP2C9*1/*1, CYP2C9*1/*2, and CYP2C9*2/*2 genotypes showed similar siponimod exposure, and participants with CYP2C9*3/*3 genotypes demonstrated an obviously decreased CL/F (about 0.3-fold) and elevated AUC_0-∞_/D (about 3.4-fold), which likely contributed to the decision of the FDA to prohibit siponimod administration in CYP2C9*3/*3 patients. Siponimod maintenance dosage recommended by the FDA was 2 mg daily in individuals with CYP2C9*1/*1, *1/*2, and *2/*2 genotypes, while it was 1 mg daily in individuals with *1/*3 and *2/*3 genotypes (Huth et al., 2019). CYP2C9 genetic polymorphism testing with subsequent dose adjustment and precaution with concomitant medications should be recommended before and during siponimod administration.

Fluconazole is one of the most clinically potent CYP3A4/CYP2C9 inhibitors. In patients with the CYP2C9*1/*1 genotype, fluconazole combination led to a siponimod AUC elevation by approximately twofold (Gardin et al., 2019b), which was consistent with the results of this study. In the wild-type genotype CYP2C9*1/*1 population, CPY2C9 showed a dominant contribution (81%). Yet in population with variant CYP2C9 alleles, as a result of the reduced enzyme activities, the CYP2C9 contribution reduced gradually to 11% in the CYP2C9*3/*3 population, meanwhile the relative contribution of CYP3A4 increased from 17% to 79% (Jin et al., 2018). This effect was considered clinically relevant. Hence, the co-administration of siponimod with a moderate CYP2C9 or strong CYP3A4 inhibitor or moderate dual inhibitor should be avoided regardless of the CYP2C9 genotype.

MBMA is a valuable quantitative pharmacology approach for model-informed drug discovery and development because it enables decision-making with a totality of evidence *via* integrating internal and external data across multiple dimensions (Upreti and Venkatakrishnan, 2019). Currently, there are several studies applying MBMA method to establish PPK models based on aggregated data (Alhadab and Brundage, 2020; Yao et al., 2021). This study presented a siponimod PPK model that integrated available pharmacokinetic data across 11 studies using the similar MBMA approach. However, due to incomplete reporting, small range of values from aggregrated data, and small number of studies, we were unable to analyze the correlation of RV, nor to characterize the effects of a range of factors impacting siponimod pharmacokinetics in MBMA process. NCA allowed to directly compare the differences in siponimod pharmacokinetic parameters across different dosage regimens, BMI, CYP2C9 genotypes, and fluconazole combination subgroups. Though NCA required frequent pharmacokinetic sampling and might be affected by potentially confounding factors, it revealed the possible influencing factors of siponimod pharmacokinetics, which was highly consistent with clinical practice, and was a powerful supplement to the results of MBMA.

## 5 Conclusion

This study successfully established a PPK model of siponimod in healthy subjects and patients with MS based on aggregated data from published clinical trials. Siponimod pharmacokinetics were adequately described by a one-compartment model with first-order absorption and elimination. A slightly lower siponimod clearance was observed in patients with MS than in healthy volunteers. The NCA results revealed that siponimod experienced an accumulation after multiple doses. BMI, CYP2C9 genetic polymorphism, and fluconazole co-administration might affect siponimod pharmacokinetics. Dosage adjustment should be considered for patients with CYP2C9*2*3/*1*3 genotypes and patients with CYP2C9 inducer/inhibitors. The PPK model and NCA results facilitated the understanding of siponimod pharmacokinetic characteristics and provided a basis for siponimod dose individualization in patients with MS.

## Data Availability

The original contributions presented in the study are included in the article/Supplementary Material, further inquiries can be directed to the corresponding authors.
