# Supplementary material for: Pharmacokinetic Characteristics of Siponimod in Healthy Volunteers and Patients With Multiple Sclerosis: Analyses of Published Clinical Trials
Source: Front Pharmacol. 2022 May 10;13:824232. doi: 10.3389/fphar.2022.824232 (PMC9127076; doi:10.3389/fphar.2022.824232)
Supplement: Supplementary file 1 [file Table1.pdf]

Supplementary Table S1 Basic model selection process

Model	Results	OFV
One-compartment model with additive error	Successful	2134.953
One-compartment model with proportional error	Successful	1560.002
**One-compartment model with mixed error**	**Successful**	**1494.675**
Two-compartment model with additive error	Successful	2018.206
Two-compartment model with proportional error	Successful	1508.045
Two-compartment model with mixed error	minimization terminated	1496.967
Three-compartment model with additive error	Failure	-
Three-compartment model with proportional error	Failure	-
Three-compartment model with mixed error	Failure	-
